# Numerical solutions of a fractional order SEIR epidemic model of measles under Caputo fractional derivative

**DOI:** 10.1371/journal.pone.0321089

**Published:** 2025-05-28

**Authors:** Nawa A. Alshammari, N. S. Alharthi, Abdulkafi Mohammed Saeed, Adnan Khan, Abdul Hamid Ganie

**Affiliations:** 1 Basic Science Department, College of Science and Theoretical Studies, Saudi Electronic University, Riyadh, Saudi Arabia; 2 Department of Mathematics, Faculty of Sciences and Arts, King Abdulaziz University, Rabigh, Saudi Arabia; 3 Department of Mathematics, College of Science, Qassim University, Buraydah, Saudi Arabia; 4 Department of Mathematics, Abdul Wali Khan University Mardan, Mardan, Pakistan; Fiji National University, FIJI

## Abstract

Measles is a highly contagious illness that can spread throughout a population based on the number of susceptible or infected individuals as well as their social dynamics within the society. The measles epidemic is thought to be controlled for the suffering population using the susceptible-exposed-infectious-recovered (SEIR) epidemic model, which depicts the direct transmission of infectious diseases. To better explain the measles epidemics, we provided a nonlinear time fractional model of the disease. The solution of SEIR is obtained by using the Caputo fractional derivative operator of order μ∈(0,1]. The Homotopy perturbation transform method (HPTM) and Yang transform decomposition methodology (YTDM) have been employed to obtain the numerical solution of the time fractional model. Obtaining numerical findings in the form of a fast-convergent series significantly improves the proposed techniques accuracy. The behaviour of the approximate series solution for several fractional orders is shown graphically which are derived through Maple. A graphic representation of the behaviours of susceptible, exposed, infected, and recovered individuals are shown at different fractional order values. Figures that depict the behaviour of the projected model are used to illustrate the developed results. Finally, the present work may help you predict the behaviour of the real-world models in the wild class with respect to the model parameters. It was found that the majority of patients who receive therapy join the recovered class when various epidemiological classes were simulated at the effect of fractional parameter μ. These approaches shows to be one of the most efficient methods to solve epidemic models and control infectious diseases.

## 1 Introduction

Medical research, public health, and health care evaluation are all grounded within the incredible field of epidemiology. These days, epidemiologists are interested in researching disease models using anonymous parameters. Mathematical epidemiology has been studying the transmission of infectious diseases since the early 20th century with the use of established mathematical models [1–4]. The deterministic and stochastic models of infectious diseases provide the researchers with important new insights. The individual populations for the deterministic models represent the various compartment-specific stages. The population transition rate stated mathematically as a derivative from one part to another depending on various parts and population transition amounts. The population as a function of time is represented by the differential equation system. In 1927, Kermack and McKendrick built a basic deterministic model that was helpful in creating more complex mathematical models of epidemics, and it is still regarded as a foundational model [5]. After the initial stage of infection, a latent period often occurs in various infectious diseases. This interval cannot be skipped while assessing the infectious stage. For this reason, it becomes sense to add an early stage into the epidemiological model. Four classifications make up the current SEIR model of disease, which corresponds to infectious diseases based on their condition. The model is made up of the following individuals: the susceptible 𝔼(χ); the exposed 𝔽(χ); the infective 𝔾(χ); and the recovered ℍ(χ). In certain cases, it is discovered that a member of the infectious class does not exhibit symptoms for some time. SEIR models are employed to model these diseases [6, 7]. Comprising, proposing, planning, executing, testing, and assessing different detection, therapy, and control programmes are among the applications of mathematical epidemiological models [8–13].

The calculus of integrals and derivatives of any arbitrary real or complex order is the focus of fractional calculus [10, 14–17]. This makes it possible to think of it as a modification of classical calculus, which is one specific example that is covered in the theory. The de l’Hospital letter to Leibniz in 1695, which asked, "What does the derivative of order $\frac{1}{3}$ or $\sqrt{2}$ of a function mean?" was the moment the notion of derivatives of non-integer order arose. The study of this field attracted the interest of mathematicians in the eighteenth and nineteenth century. Famous scientist Abel became the first to investigate tautochrone problems implicitly using fractional calculus in 1823 [19]. After that, a number of fundamental papers have been published on different facets of fractional calculus [18,20–22]. A comprehensive collection of relevant findings, including case studies and examples, can be found in [23]. Additionally, there exists an extensive amount of work in the background literature pertaining to the exact and approximate solutions of fractional differential equations (FDEs) of the Riemann Liouville and Caputo types, as well as non-integer derivatives concerning polynomial products, non-integer derivatives and non-integer powers of operators, and boundary value problems (see, for example, [24–28]). Researchers have recently focused a lot of attention on fractional calculus, and several facets of the topic are being explored for research. This is an illustration of the fractional derivative’s importance as a tool for understanding the dynamic behaviour of many physical systems.

The use of fractional calculus in a number of scientific and engineering fields is noteworthy and highly significant [29–31]. More specifically, the biological mechanisms underlying a number of diseases have been studied using fractional framework [32–35]. It has been shown that fractional calculus is useful for modeling a wide range of processes and has many applications. The nonlocal properties of this differential operator are its strongest strength, which are absent within integer order differential operators. FDEs are unique in that they may describe the memory and transmission properties of many different mathematical models. It is a known reality that models with fractional orders are more useful and realistic than those with integer orders. In these models, the fractional order derivative yields a higher degree of freedom. Strong tools for controlling the dynamic behaviour of different biomaterials and systems are arbitrary order derivatives [27]. These models most important trait is their global (nonlocal) properties, which are absent within classical order models [36]. However, there is also an increasing amount of work on dynamic fractional differential systems that is related to modern mathematics and is focused on several scientific domains such as control theory, chemistry, and physics. Fractional calculus may be interesting because it provides a more detailed description of potential uncertainties in the dynamic model due to the numerical value of the fraction parameter. In particular, fractional calculus has been applied in a number of real-world scenarios recently [37–40. However, there is currently an expanding collection of mathematical literature on fractional differ-integral calculus that can successfully confirm support for studies conducted in other relevant fields [41–42].

In reality, the analytical analysis of a fractional derivative model is one of its difficulties. In fact, this restricts attention to the fractional model’s dynamics. To the best of our knowledge, there are no pertinent publications in the available literature that address the process of deriving analytical solutions for the measles model. Furthermore, not much research has been done to examine the dynamics of the SEIR model using non-integer derivatives. Overall, the literature suggests that more investigation and study are needed to better understand and study the SEIR model phenomenon. Therefore, we present a comprehensive analysis of the intricate phenomena of SEIR model to understand the transmission route of this infectious disease. Powerful techniques such as YTDM and HPTM are produced by combining the adomian decomposition method (ADM), homotopy perturbation method (HPM), and Yang transform. Adomain polynomials and He’s polynomials are used to decompose the nonlinear terms after the differential equations are transformed into algebraic equations with the aid of Yang transform. Both deterministic and stochastic differential equation systems can be solved effectively with these computational methods. More specifically, it can be applied to a system of fractional order, classical, linear, and nonlinear ordinary and partial differential equations. The structure of the article is as follows: The standard SEIR model is described in Sect 2. We give some fundamental definitions of FC in Sect 3. We created the HPTM for differential equations of any order in Sect 4. We created the YTDM for differential equations of any order in Sect 5. We discussed the convergence analysis of the suggested techniques in Sect 6. We solved the SEIR epidemic model in Sect 7 using the suggested methods. Discussion and numerical simulation are provided in Sect 8. Sect 9 concludes with some final remarks.

## 2 Model descriptions

**Fig 1:**
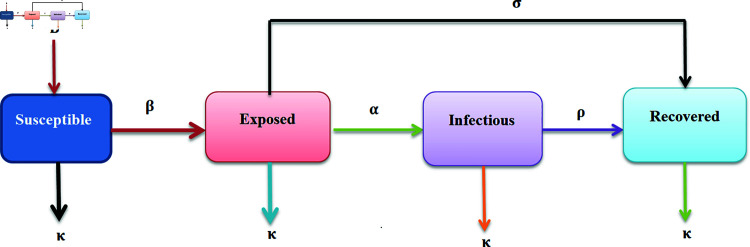
Flow diagram of SEIR model.

To explain the spread dynamics of measles, we design a deterministic, compartmental mathematical model. The population is constantly interacting and mimics the demographics of a normal developing nation while exploring dynamics that are growing exponentially. The model equations [10] are described by classifying the total population (𝐍) into four categories: 𝔼,𝔽,𝔾, and ℍ, which stand for susceptible, exposed, infected, and recovered population, respectively. The flow chart of these four classes are shown in Fig 1.

$$\frac{d𝔼}{d\chi }=ℬ-\beta 𝔼(\chi )𝔾(\chi )-\kappa 𝔼(\chi ),\;\frac{d𝔽}{d\chi }=\beta 𝔼(\chi )𝔾(\chi )-(\sigma +\kappa +\alpha )𝔽(\chi ),\;\frac{d𝔾}{d\chi }=\alpha 𝔽(\chi )-(\rho +\kappa )𝔾(\chi ),\;\frac{dℍ}{d\chi }=\rho 𝔾(\chi )+\sigma 𝔽(\chi )-\kappa ℍ(\chi ),$$ (1)

The class 𝔼 of susceptible is decreased with infected individuals at a rate β; the rate at which new immigrants or births occur is ℬ; and the rate at which natural death occurs is κ. The exposed individuals in 𝔽 are formed by interaction with infected persons at a rate of β; these individuals break into the infected class at a rate of α, become diminished at a rate of κ, and are decreased at a rate of σ through testing and measles therapy. At a speed α, the class of infected individual 𝔾 is formed from the exposed individual. It gets weaker at a rate κ and reduced by infection recovery at a rate ρ. This creates a class ℍ that is fully protected from individual disease. According to [10], the natural death at a rate κ is diminished by ℍ recovered individual.

The model’s fractional order expansion was first investigated in [43]. Although at a slower rate, they exhibit the realistic biphasic decrease behaviour of disease infection. The fractional SEIR system is the new differential equation system that is represented as follows.

$$D_{\chi }^{\mu_{1}}𝔼(\chi )=ℬ-\beta 𝔼(\chi )𝔾(\chi )-\kappa 𝔼(\chi ),\;D_{\chi }^{\mu_{2}}𝔽(\chi )=\beta 𝔼(\chi )𝔾(\chi )-(\sigma +\kappa +\alpha )𝔽(\chi ),\;D_{\chi }^{\mu_{3}}𝔾(\chi )=\alpha 𝔽(\chi )-(\rho +\kappa )𝔾(\chi ),\;D_{\chi }^{\mu_{4}}ℍ(\chi )=\rho 𝔾(\chi )+\sigma 𝔽(\chi )-\kappa ℍ(\chi ),\;$$ (2)

The above model becomes the classical epidemic model for $\mu_{1}=\mu_{2}=\mu_{3}=\mu_{4}=1$.

## 3 Basic concept

To provide more insight and elucidate the methods of solution, we will draw attention to a helpful concept pertaining to fractional calculus.

### 3.1 Definition

The Caputo fractional operator is as [44]

$$D_{\chi }^{\mu }𝔼(\chi )=\frac{1}{\Gamma (k-\mu )}\int_{0}^{\chi }(\chi -\gamma {)}^{k-\mu -1}{𝔼}^{\left(k\right)}(\gamma )d\gamma ,\;k-1<\mu \leq k,\;k\in N.$$ (3)

### 3.2 Definition

The yang transform (YT) of the function below is as [45]

$$Y\{𝔼(\chi )\}=𝒥(\varsigma )=\int_{0}^{\infty }e^{\frac{-\chi }{\varsigma }}𝔼(\chi )d\chi ,\;\;\chi >0,$$ (4)

with ς demonstrate the transform variable.

Some basic properties of YT are as

$$Y[1]=\varsigma ,\;Y[\chi ]=\varsigma^{2},\;Y[\chi^{k}]=\Gamma (k+1)\varsigma^{k+1}.$$ (5)

having inverse YT as

$$Y^{-1}\{ℱ(\varsigma )\}=𝔼(\chi ).$$ (6)

### 3.3 Definition

The YT of the function having n-times derivative is as[45]

$$Y\{{𝔼}^{n}(\chi )\}=\frac{ℱ(\varsigma )}{\varsigma^{n}}-\sum_{k=0}^{n-1}\frac{{𝔼}^{k}(0)}{\varsigma^{n-k-1}},\;\;\forall \;\;n=1,2,3,\cdots$$ (7)

### 3.4 Definition

The YT of the function with non-integer derivative is as [45]

$$Y\{{𝔼}^{\mu }(\chi )\}=\frac{ℱ(\varsigma )}{\varsigma^{\mu }}-\sum_{k=0}^{n-1}\frac{{𝔼}^{k}(0)}{\varsigma^{\mu -(k+1)}},\;\;0<\mu \leq n.$$ (8)

## 4 Analysis of the HPTM

Assume the general nonlinear fractional differential equation as

$$D_{\chi }^{\mu }𝔼(\chi )=ℐ(𝔼(\chi ))+𝒥(𝔼(\chi )),\;\;0<\mu \leq 1,$$ (9)

having


𝔼(0)=ξ(ϑ).

By using the YT

$$Y[D_{\chi }^{\mu }𝔼(\chi )]=Y[ℐ(𝔼(\chi ))+𝒥(𝔼(\chi ))],$$ (10)

$$\frac{1}{\varsigma^{\mu }}\{ℱ(\varsigma )-\varsigma 𝔼(0)\}=Y[ℐ(𝔼(\chi ))+𝒥(𝔼(\chi ))].$$ (11)

After we get

$$ℱ(\varsigma )=\varsigma 𝔼(0)+\varsigma^{\mu }Y[ℐ(𝔼(\chi ))+𝒥(𝔼(\chi ))].$$ (12)

On utilizing the inverse YT

$$𝔼(\chi )=𝔼(0)+Y^{-1}[\varsigma^{\mu }Y[ℐ(𝔼(\chi ))+𝒥(𝔼(\chi ))]].$$ (13)

In terms of HPM, we may get

$$𝔼(\chi )=\sum_{k=0}^{\infty }\epsilon^{k}{𝔼}_{k}(\chi ),$$ (14)

with ϵ∈[0,1].

Let

$$𝒥(𝔼(\chi ))=\sum_{k=0}^{\infty }\epsilon^{k}H_{n}(𝔼),$$ (15)

with

$$H_{n}({𝔼}_{0},{𝔼}_{1},...,{𝔼}_{n})=\frac{1}{\Gamma (n+1)}D_{\epsilon }^{k}{\left[𝒥\left(\sum_{k=0}^{\infty }\epsilon^{i}{𝔼}_{i}\right)\right]}_{\epsilon =0},$$ (16)

where $D_{\epsilon }^{k}=\frac{\partial^{k}}{\partial \epsilon^{k}}.$

By switching Eq (14) and Eq (15) in Eq (13), we may get

$$\sum_{k=0}^{\infty }\epsilon^{k}{𝔼}_{k}(\chi )=𝔼(0)+\epsilon \times \left(Y^{-1}\left[\varsigma^{\mu }Y\{ℐ\sum_{k=0}^{\infty }\epsilon^{k}{𝔼}_{k}(\chi )+\sum_{k=0}^{\infty }\epsilon^{k}H_{k}(𝔼)\}\right]\right).$$ (17)

Similarly,

$$\epsilon^{0}:{𝔼}_{0}(\chi )=𝔼(0),\;\epsilon^{1}:{𝔼}_{1}(\chi )=Y^{-1}\left[\varsigma^{\mu }Y(ℐ({𝔼}_{0}(\chi ))+H_{0}(𝔼))\right],\;\epsilon^{2}:{𝔼}_{2}(\chi )=Y^{-1}\left[\varsigma^{\mu }Y(ℐ({𝔼}_{1}(\chi ))+H_{1}(𝔼))\right],\;.\;.\;.\;\epsilon^{k}:{𝔼}_{k}(\chi )=Y^{-1}\left[\varsigma^{\mu }Y(ℐ({𝔼}_{k-1}(\chi ))+H_{k-1}(𝔼))\right],\;k>0,k\in N.$$ (18)

Finally, the approximate solution is derived as

$$𝔼(\chi )=\lim_{M\to \infty }\sum_{k=1}^{M}{𝔼}_{k}(\chi ).$$ (19)

## 5 Analysis of the YTDM

Assume the general nonlinear fractional differential equation as

$$D_{\chi }^{\mu }𝔼(\chi )=ℐ(\chi )+𝒥(\chi ),\;\;0<\mu \leq 1,$$ (20)

having


𝔼(0)=ξ(ϑ).

By using the YT

$$Y[D_{\chi }^{\mu }𝔼(\chi )]=Y[ℐ(\chi )+𝒥(\chi )],\;\frac{1}{\varsigma^{\mu }}\{ℱ(\varsigma )-\varsigma 𝔼(0)\}=Y[ℐ(\chi )+𝒥(\chi )].\;$$ (21)

After we get

$$ℱ(\varsigma )=\varsigma 𝔼(0)+\varsigma^{\mu }Y[ℐ(\chi )+𝒥(\chi )].$$ (22)

On utilizing the inverse YT

$$𝔼(\chi )=𝔼(0)+Y^{-1}[\varsigma^{\mu }Y[ℐ(\chi )+𝒥(\chi )].$$ (23)

We may get the series form solution as

$$𝔼(\chi )=\sum_{m=0}^{\infty }{𝔼}_{m}(\chi ).$$ (24)

The nonlinear term is broken down as

$$𝒥(\chi )=\sum_{m=0}^{\infty }{𝒜}_{m},$$ (25)

with

$${𝒜}_{m}=\frac{1}{m!}{\left[\frac{\partial^{m}}{\partial \ell^{m}}\left\{𝒥\left(\sum_{k=0}^{\infty }\ell_{k}^{k},\sum_{k=0}^{\infty }\ell^{k}\chi_{k}\right)\right\}\right]}_{\ell =0}.$$ (26)

By switching Eq (24) and Eq (25) in Eq (23), we may get

$$\sum_{m=0}^{\infty }{𝔼}_{m}(\chi )=𝔼(0)+Y^{-1}\varsigma^{\mu }\left[Y\left\{ℐ(\sum_{m=0}^{\infty }\chi_{m})+\sum_{m=0}^{\infty }{𝒜}_{m}\right\}\right].$$ (27)

Similarly,

$${𝔼}_{0}(\chi )=𝔼(0),$$ (28)

$${𝔼}_{1}(\chi )=Y^{-1}\left[\varsigma^{\mu }Y\{ℐ(\chi_{0})+{𝒜}_{0}\}\right].$$

Finally, the approximate solution is derived as


$${𝔼}_{m+1}(\chi )=Y^{-1}\left[\varsigma^{\mu }Y\{ℐ(\chi_{m})+{𝒜}_{m}\}\right].$$

## 6 Convergence analysis

Here we discuss the convergence analysis of the proposed approaches.

### 6.1 Theorem

Let us assume that the precise solution of (9) is 𝔼(χ) and let 𝔼(χ), ${𝔼}_{n}(\chi )\in H$ and ζ∈(0,1), where H symbolizes the Hilbert space. The solution achieved $\sum_{q=0}^{\infty }{𝔼}_{q}(\chi )$ will converge 𝔼(χ) if ${𝔼}_{q}(\chi )\leq {𝔼}_{q-1}(\chi )\;\;\forall q>A$, i.e., for any ω>0∃A>0, such that $||{𝔼}_{q+n}(\chi )||\leq \beta$, ∀m,n∈N.

Proof. We take a sequence of $\sum_{q=0}^{\infty }{𝔼}_{q}(\chi ).$

$$\Theta_{0}(\chi )={𝔼}_{0}(\chi ),\;\Theta_{1}(\chi )={𝔼}_{0}(\chi )+{𝔼}_{1}(\chi ),\;\Theta_{2}(\chi )={𝔼}_{0}(\chi )+{𝔼}_{1}(\chi )+{𝔼}_{2}(\chi ),\;\Theta_{3}(\chi )={𝔼}_{0}(\chi )+{𝔼}_{1}(\chi )+{𝔼}_{2}(\chi )+{𝔼}_{3}(\chi ),\;\vdots \;\Theta_{q}(\chi )={𝔼}_{0}(\chi )+{𝔼}_{1}(\chi )+{𝔼}_{2}(\chi )+\cdots +{𝔼}_{q}(\chi ),\;$$ (29)

We must demonstrate that $\Theta_{q}(\chi )$ forms a “Cauchy sequence” in order to achieve the desired outcome. Additionally, let’s take

$$||\Theta_{q+1}(\chi )-\Theta_{q}(\chi )||=||{𝔼}_{q+1}(\chi )||\leq \zeta ||{𝔼}_{q}(\chi )||\leq \zeta^{2}||{𝔼}_{q-1}(\chi )||\leq \zeta^{3}||{𝔼}_{q-2}(\chi )||\cdots \;\leq \zeta_{q+1}||{𝔼}_{0}(\chi )||.$$ (30)

For q,n∈N, we have

$$||\Theta_{q}(\chi )-\Theta_{n}(\chi )||=||{𝔼}_{q+n}(\chi )||=||\Theta_{q}(\chi )-\Theta_{q-1}(\chi )+(\Theta_{q-1}(\chi )-\Theta_{q-2}(\chi ))\;+(\Theta_{q-2}(\chi )-\Theta_{q-3}(\chi ))+\cdots +(\Theta_{n+1}(\chi )-\Theta_{n}(\chi ))||\;\leq ||\Theta_{q}(\chi )-\Theta_{q-1}(\chi )||+||(\Theta_{q-1}(\chi )-\Theta_{q-2}(\chi ))||\;+||(\Theta_{q-2}(\chi )-\Theta_{q-3}(\chi ))||+\cdots +||(\Theta_{n+1}(\chi )-\Theta_{n}(\chi ))||\;\leq \zeta^{q}||{𝔼}_{0}(\chi )||+\zeta^{q-1}||{𝔼}_{0}(\chi )||+\cdots +\zeta^{q+1}||{𝔼}_{0}(\chi )||\;=||{𝔼}_{0}(\chi )||(\zeta^{q}+\zeta^{q-1}+\zeta^{q+1})\;=||{𝔼}_{0}(\chi )||\frac{1-\zeta^{q-n}}{1-\zeta^{q+1}}\zeta^{n+1}.$$ (31)

As 0<ζ<1, and ${𝔼}_{0}(\chi )$ are bound, so take $\beta =1-\zeta /(1-\zeta_{q-n})\zeta^{n+1}||{𝔼}_{0}(\chi )||$, and we get

$$||{𝔼}_{q+n}(\chi )||\leq \beta ,\forall q,n\in N.$$ (32)

Hence, $\{{𝔼}_{q}(\chi ){\}}_{q=0}^{\infty }$ makes a “Cauchy sequence” in H. It proves that the sequence $\{{𝔼}_{q}(\chi ){\}}_{q=0}^{\infty }$ is a convergent sequence with the limit $\lim_{q\to \infty }{𝔼}_{q}(\chi )=𝔼(\chi )$ for ∃𝔼(χ)∈ℐ which complete the proof.

### 6.2 Theorem

Assuming that $\sum_{h=0}^{k}{𝔼}_{h}(\chi )$ is finite and 𝔼(χ) reflect the series solution that was establish. Considering ζ>0 such that $\left||{𝔼}_{h+1}(\chi )||\leq ||{𝔼}_{h}(\chi )|\right|$, the maximum absolute error is presumed by the resultant relation.

$$||𝔼(\chi )-\sum_{h=0}^{k}{𝔼}_{h}(\chi )||<\frac{\zeta^{k+1}}{1-\zeta }||{𝔼}_{0}(\chi )||.$$ (33)

Proof. Assume $\sum_{h=0}^{k}{𝔼}_{h}(\chi )$ is finite which indicates that $\sum_{h=0}^{k}{𝔼}_{h}(\chi )<\infty$.

Let us consider

$$||𝔼(\chi )-\sum_{h=0}^{k}{𝔼}_{h}(\chi )||=||\sum_{h=k+1}^{\infty }{𝔼}_{h}(\chi )||\;\leq \sum_{h=k+1}^{\infty }||{𝔼}_{h}(\chi )||\;\leq \sum_{h=k+1}^{\infty }\zeta^{h}||{𝔼}_{0}(\chi )||\;\leq \zeta^{k+1}(1+\zeta +\zeta^{2}+\cdots )||{𝔼}_{0}(\chi )||\;\leq \frac{\zeta^{k+1}}{1-\zeta }||{𝔼}_{0}(\chi )||.$$ (34)

which complete the proof of theorem.

### 6.3 Theorem

The result of (20) is unique when $0<(\Pi_{1}+\Pi_{2})(\frac{\chi^{\mu }}{\Gamma (\mu +1)})<1.$

Proof: Let H=(C[J],||.||) with the norm $\left||𝔼(\chi )||={max}_{\chi \in J}|𝔼(\chi )\right|$ is Banach space,∀ continuous function on *J*. Let I:H→H is a non-linear mapping, where


$${𝔼}_{l+1}^{C}={𝔼}_{0}^{C}+Y^{-1}[\varsigma^{\mu }Y[ℐ({𝔼}_{l}(\chi ))+𝒥({𝔼}_{l}(\chi ))]],\;\;l\geq 0.$$

Suppose that $\left|ℐ(𝔼)-ℐ({𝔼}^{*})|<\Pi_{1}|𝔼-{𝔼}^{*}\right|$ and $\left|𝒥(𝔼)-𝒥({𝔼}^{*})|<\Pi_{2}|𝔼-{𝔼}^{*}\right|$, where 𝔼:=𝔼(χ) and ${𝔼}^{*}:={𝔼}^{*}(\chi )$ are are two different function values and $\Pi_{1}$,$\Pi_{2}$ are Lipschitz constants.

$$\left||I𝔼-I{𝔼}^{*}||\leq max_{t\in J}|Y^{-1}[\varsigma^{\mu }Y[ℐ(𝔼)-ℐ({𝔼}^{*})\;+\varsigma^{\mu }Y[𝒥(𝔼)-𝒥({𝔼}^{*})]|]\;\leq max_{\chi \in J}[\Pi_{1}Y^{-1}[\varsigma^{\mu }Y[|𝔼-{𝔼}^{*}|]]\;+\Pi_{2}Y^{-1}[\varsigma^{\mu }Y[|𝔼-{𝔼}^{*}|]]]\;\leq max_{t\in J}(\Pi_{1}+\Pi_{2})\left[Y^{-1}[\varsigma^{\mu }Y|𝔼-{𝔼}^{*}|]\right]\;\leq (\Pi_{1}+\Pi_{2})\left[Y^{-1}[\varsigma^{\mu }Y||𝔼-{𝔼}^{*}||]\right]\;=(\Pi_{1}+\Pi_{2})(\frac{\chi^{\mu }}{\Gamma (\mu +1)})||𝔼-{𝔼}^{*}|\right|$$ (35)

I is contraction as $0<(\Pi_{1}+\Pi_{2})(\frac{\chi^{\mu }}{\Gamma (\mu +1)})<1$. From Banach fixed point theorem the result of (19) is unique.

### 6.4 Theorem

The result of (20) is convergent.

Proof: Let ${𝔼}_{m}=\sum_{r=0}^{m}{𝔼}_{r}(\chi )$. To show that ${𝔼}_{m}$ is a Cauchy sequence in H. Let,

$$\left||{𝔼}_{m}-{𝔼}_{n}||=max_{\chi \in J}|\sum_{r=n+1}^{m}{𝔼}_{r}|,\;\;n=1,2,3,\cdots \;\leq max_{\chi \in J}\left|Y^{-1}\left[\varsigma^{\mu }Y\left[\sum_{r=n+1}^{m}(ℐ({𝔼}_{r-1})+𝒥({𝔼}_{r-1}))\right]\right]\right|\;=max_{\chi \in J}\left|Y^{-1}\left[\varsigma^{\mu }Y\left[\sum_{r=n+1}^{m-1}(ℐ({𝔼}_{r})+𝒥({𝔼}_{r}))\right]\right]\right|\;\leq max_{\chi \in J}|Y^{-1}[\varsigma^{\mu }Y[(ℐ({𝔼}_{m-1})-ℐ({𝔼}_{n-1})+𝒥({𝔼}_{m-1})-𝒥({𝔼}_{n-1}))]]|\;\leq \Pi_{1}max_{\chi \in J}|Y^{-1}[\varsigma^{\mu }Y[(ℐ({𝔼}_{m-1})-ℐ({𝔼}_{n-1}))]]|\;+\Pi_{[}]2max_{\chi \in J}|Y^{-1}[\varsigma^{\mu }Y[(𝒥({𝔼}_{m-1})-𝒥({𝔼}_{n-1}))]]|\;=(\Pi_{1}+\Pi_{2})(\frac{\chi^{\mu }}{\Gamma (\mu +1)})||{𝔼}_{m-1}-{𝔼}_{n-1}|\right|$$ (36)

Let *m* = *n* + 1, then

$$||{𝔼}_{n+1}-{𝔼}_{n}||\leq \Pi ||{𝔼}_{n}-{𝔼}_{n-1}||\leq \Pi^{2}||{𝔼}_{n-1}{𝔼}_{n-2}||\leq \cdots \leq \Pi^{n}||{𝔼}_{1}-{𝔼}_{0}||,$$ (37)

where $\Pi =(\Pi_{1}+\Pi_{2})(\frac{\chi^{\mu }}{\Gamma (\mu +1)})$. Similarly, we have

$$||{𝔼}_{m}-{𝔼}_{n}||\leq ||{𝔼}_{n+1}-{𝔼}_{n}||+||{𝔼}_{n+2}{𝔼}_{n+1}||+\cdots +||{𝔼}_{m}-{𝔼}_{m-1}||,\;(\Pi^{n}+\Pi^{n+1}+\cdots +\Pi^{m-1})||{𝔼}_{1}-{𝔼}_{0}||\;\leq \Pi^{n}\left(\frac{1-\Pi^{m-n}}{1-\Pi }\right)||{𝔼}_{1}||,$$ (38)

As 0<Π<1, we get $1-\Pi^{m-n}<1$. Therefore,

$$||{𝔼}_{m}-{𝔼}_{n}||\leq \frac{\Pi^{n}}{1-\Pi }max_{\chi \in J}||{𝔼}_{1}||.$$ (39)

Since $||{𝔼}_{1}||<\infty ,\;\;||{𝔼}_{m}\;-\;{𝔼}_{n}||\to 0$ when n→∞. Thus, ${𝔼}_{m}$ is a Cauchy sequence in H, indicating that the series ${𝔼}_{m}$ is convergent.

## 7 Applications

### 7.1 Example

Assume the fractional epidemic model (2) with below initial conditions.


$$𝔼(0)=n_{1},𝔽(0)=n_{2},𝔾(0)=n_{3}\;\;and\;\;ℍ(0)=n_{4}.$$

By using the YT

$$Y\left(\frac{\partial^{\mu_{1}}𝔼}{\partial \chi^{q}}\right)=Y(ℬ-\beta 𝔼(\chi )𝔾(\chi )-\kappa 𝔼(\chi )),\;Y\left(\frac{\partial^{\mu_{2}}𝔽}{\partial \chi^{q}}\right)=Y(\beta 𝔼(\chi )𝔾(\chi )-(\sigma +\kappa +\alpha )𝔽(\chi )),\;Y\left(\frac{\partial^{\mu_{3}}𝔾}{\partial \chi^{q}}\right)=Y(\alpha 𝔽(\chi )-(\rho +\kappa )𝔾(\chi )),\;Y\left(\frac{\partial^{\mu_{4}}ℍ}{\partial \chi^{q}}\right)=Y(\rho 𝔾(\chi )+\sigma 𝔽(\chi )-\kappa ℍ(\chi )).\;$$ (40)

After we get

$$\frac{1}{\varsigma^{\mu_{1}}}\{ℱ(\varsigma )-\varsigma 𝔼(0)\}=Y(ℬ-\beta 𝔼(\chi )𝔾(\chi )-\kappa 𝔼(\chi )),\;\frac{1}{\varsigma^{\mu_{2}}}\{ℱ(\varsigma )-\varsigma 𝔽(0)\}=Y(\beta 𝔼(\chi )𝔾(\chi )-(\sigma +\kappa +\alpha )𝔽(\chi )),\;\frac{1}{\varsigma^{\mu_{3}}}\{ℱ(\varsigma )-\varsigma 𝔾(0)\}=Y(\alpha 𝔽(\chi )-(\rho +\kappa )𝔾(\chi )),\;\frac{1}{\varsigma^{\mu_{4}}}\{ℱ(\varsigma )-\varsigma ℍ(0)\}=Y(\rho 𝔾(\chi )+\sigma 𝔽(\chi )-\kappa ℍ(\chi )),\;$$ (41)

$$ℱ(\varsigma )=\varsigma 𝔼(0)+\varsigma^{\mu_{1}}(ℬ-\beta 𝔼(\chi )𝔾(\chi )-\kappa 𝔼(\chi )),\;ℱ(\varsigma )=\varsigma 𝔽(0)+\varsigma^{\mu_{2}}(\beta 𝔼(\chi )𝔾(\chi )-(\sigma +\kappa +\alpha )𝔽(\chi )),\;ℱ(\varsigma )=\varsigma 𝔾(0)+\varsigma^{\mu_{3}}(\alpha 𝔽(\chi )-(\rho +\kappa )𝔾(\chi )),\;ℱ(\varsigma )=\varsigma ℍ(0)+\varsigma^{\mu_{4}}(\rho 𝔾(\chi )+\sigma 𝔽(\chi )-\kappa ℍ(\chi )),$$ (42)

On utilizing the inverse YT

$$𝔼(\chi )=𝔼(0)+Y^{-1}[\varsigma^{\mu_{1}}Y(ℬ)]+Y^{-1}[\varsigma^{\mu_{1}}\left\{Y(-\beta 𝔼(\chi )𝔾(\chi )-\kappa 𝔼(\chi ))\right\}],\;𝔽(\chi )=𝔽(0)+Y^{-1}[\varsigma^{\mu_{2}}\left\{Y(\beta 𝔼(\chi )𝔾(\chi )-(\sigma +\kappa +\alpha )𝔽(\chi ))\right\}],\;𝔾(\chi )=𝔾(0)+Y^{-1}[\varsigma^{\mu_{3}}\left\{Y(\alpha 𝔽(\chi )-(\rho +\kappa )𝔾(\chi ))\right\}],\;ℍ(\chi )=ℍ(0)+Y^{-1}[\varsigma^{\mu_{4}}\left\{Y(\rho 𝔾(\chi )+\sigma 𝔽(\chi )-\kappa ℍ(\chi ))\right\}],$$ (43)

$$𝔼(\chi )=n_{1}+Y^{-1}[\varsigma^{\mu_{1}}Y(ℬ)]+Y^{-1}[\varsigma^{\mu_{1}}\left\{Y(-\beta 𝔼(\chi )𝔾(\chi )-\kappa 𝔼(\chi ))\right\}],\;𝔽(\chi )=n_{2}+Y^{-1}[\varsigma^{\mu_{2}}\left\{Y(\beta 𝔼(\chi )𝔾(\chi )-(\sigma +\kappa +\alpha )𝔽(\chi ))\right\}],\;𝔾(\chi )=n_{3}+Y^{-1}[\varsigma^{\mu_{3}}\left\{Y(\alpha 𝔽(\chi )-(\rho +\kappa )𝔾(\chi ))\right\}],\;ℍ(\chi )=n_{4}+Y^{-1}[\varsigma^{\mu_{4}}\left\{Y(\rho 𝔾(\chi )+\sigma 𝔽(\chi )-\kappa ℍ(\chi ))\right\}].\;$$ (44)

In terms of HPM, we may get

$$\sum_{k=0}^{\infty }\epsilon^{k}{𝔼}_{k}(\chi )=n_{1}+ℬ\frac{\chi^{\mu_{1}}}{\Gamma (\mu_{1}+1)}+\epsilon [Y^{-1}[\varsigma^{\mu_{1}}Y[-\beta (\sum_{k=0}^{\infty }\epsilon^{k}H_{k}^{1}(𝔼))-\;\kappa (\sum_{k=0}^{\infty }\epsilon^{k}{𝔼}_{k}(\chi ))]]],\;\sum_{k=0}^{\infty }\epsilon^{k}{𝔽}_{k}(\chi )=n_{2}+\epsilon [Y^{-1}[\varsigma^{\mu_{2}}Y[\beta (\sum_{k=0}^{\infty }\epsilon^{k}H_{k}^{1}(𝔼))-(\sigma +\kappa +\alpha )(\sum_{k=0}^{\infty }\epsilon^{k}{𝔽}_{k}(\chi ))]]],\;\sum_{k=0}^{\infty }\epsilon^{k}{𝔾}_{k}(\chi )=n_{3}+\epsilon [Y^{-1}[\varsigma^{\mu_{3}}Y[\alpha (\sum_{k=0}^{\infty }\epsilon^{k}{𝔽}_{k}(\chi ))-(\rho +\kappa )(\sum_{k=0}^{\infty }\epsilon^{k}{𝔾}_{k}(\chi ))]]],\;\sum_{k=0}^{\infty }\epsilon^{k}{ℍ}_{k}(\chi )=n_{4}+\epsilon [Y^{-1}[\varsigma^{\mu_{4}}Y[\rho (\sum_{k=0}^{\infty }\epsilon^{k}{𝔾}_{k}(\chi ))-\sigma (\sum_{k=0}^{\infty }\epsilon^{k}{𝔽}_{k}(\chi ))-\;\kappa (\sum_{k=0}^{\infty }\epsilon^{k}{ℍ}_{k}(\chi ))]]],\;$$ (45)

For numerical results, we used the following (Table 1) values of parameters are considered from [10]. By comparing the ϵ coefficients, we may get


$$\epsilon^{0}:{𝔼}_{0}(\chi )=600+0.32\frac{\chi^{\mu_{1}}}{\Gamma (\mu_{1}+1)},$$

$$\epsilon^{0}:{𝔽}_{0}(\chi )=250,$$

$$\epsilon^{0}:{𝔾}_{0}(\chi )=100,$$

$$\epsilon^{0}:{ℍ}_{0}(\chi )=50,$$

$$\epsilon^{1}:{𝔼}_{1}(\chi )=-720\frac{\chi^{\mu_{1}}}{\Gamma (\mu_{1}+1)}-0.32\frac{\chi^{2\mu_{1}}}{\Gamma (2\mu_{1}+1)},$$

$$\epsilon^{1}:{𝔽}_{1}(\chi )=485\frac{\chi^{\mu_{2}}}{\Gamma (\mu_{2}+1)}+0.32\frac{\chi^{\mu_{1}+\mu_{2}}}{\Gamma (\mu_{1}+\mu_{2}+1)},$$

$$\epsilon^{1}:{𝔾}_{1}(\chi )=-37.5\frac{\chi^{\mu_{3}}}{\Gamma (\mu_{3}+1)},$$

$$\epsilon^{1}:{ℍ}_{1}(\chi )=72.5\frac{\chi^{\mu_{4}}}{\Gamma (\mu_{4}+1)},$$

$$\epsilon^{2}:{𝔼}_{2}(\chi )=205\frac{\chi^{\mu_{1}+\mu_{3}}}{\Gamma (\mu_{1}+\mu_{3}+1)}+0.12\frac{\chi^{\mu_{1}+\mu_{3}}}{\Gamma (\mu_{1}+1)\Gamma (\mu_{3}+1)}\frac{\chi^{2\mu_{1}+\mu_{3}}}{\Gamma (2\mu_{1}+\mu_{3}+1)}+864$$

$$\frac{\chi^{2\mu_{1}}}{\Gamma (2\mu_{1}+1)}+0.384\frac{\chi^{3\mu_{1}}}{\Gamma (3\mu_{1}+1)},$$

$$\epsilon^{2}:{𝔽}_{2}(\chi )=-205\frac{\chi^{\mu_{2}+\mu_{3}}}{\Gamma (\mu_{2}+\mu_{3}+1)}-0.12\frac{\chi^{\mu_{1}+\mu_{3}}}{\Gamma (\mu_{1}+1)\Gamma (\mu_{3}+1)}\frac{\chi^{\mu_{1}+\mu_{2}+\mu_{3}}}{\Gamma (\mu_{1}+\mu_{2}+\mu_{3}+1)}-$$

$$720\frac{\chi^{\mu_{1}+\mu_{2}}}{\Gamma (\mu_{1}+\mu_{2}+1)}-0.32$$

$$\frac{\chi^{2\mu_{1}+\mu_{2}}}{\Gamma (2\mu_{1}+\mu_{2}+1)}-223.1\frac{\chi^{2\mu_{2}}}{\Gamma (2\mu_{2}+1)},$$

$$\epsilon^{2}:{𝔾}_{2}(\chi )=4.85\frac{\chi^{\mu_{2}+\mu_{3}}}{\Gamma (\mu_{2}+\mu_{3}+1)}+0.0032\frac{\chi^{\mu_{1}+\mu_{2}+\mu_{3}}}{\Gamma (\mu_{1}+\mu_{2}+\mu_{3}+1)}+15\frac{\chi^{2\mu_{3}}}{\Gamma (2\mu_{3}+1)},$$

$$\epsilon^{2}:{ℍ}_{2}(\chi )=-7.5\frac{\chi^{\mu_{3}+\mu_{4}}}{\Gamma (\mu_{3}+\mu_{4}+1)}+121.5\frac{\chi^{\mu_{2}+\mu_{4}}}{\Gamma (\mu_{2}+\mu_{4}+1)}+0.08\frac{\chi^{\mu_{1}+\mu_{2}+\mu_{4}}}{\Gamma (\mu_{1}+\mu_{2}+\mu_{4}+1)}$$

$$-14.5\frac{\chi^{2\mu_{4}}}{\Gamma (2\mu_{4}+1)},$$

⋮

**Table 1:** Parameter values of SEIR measles model.

Parameter	Values	Description
ℬ	0.32	Birth or immigration at a rate
*n*_1_	600	Initial population of 𝔼(χ)
*n*_2_	250	Initial population of 𝔽(χ)
*n*_3_	100	Initial population of 𝔾(χ)
*n*_4_	50	Initial population of ℍ(χ)
β	0.01	Infected individual rate
ρ	0.2	Recovery from infection rate
κ	0.2	Natural death rate
σ	0.25	Measles therapy rate
α	0.01	Infected class rate

Finally, the approximate solution is derived as


$$𝔼(\chi )={𝔼}_{0}(\chi )+{𝔼}_{1}(\chi )+{𝔼}_{2}(\chi )+\cdots$$

$$𝔼(\chi )=600+0.32\frac{\chi^{\mu_{1}}}{\Gamma (\mu_{1}+1)}-720\frac{\chi^{\mu_{1}}}{\Gamma (\mu_{1}+1)}-0.32\frac{\chi^{2\mu_{1}}}{\Gamma (2\mu_{1}+1)}+205\frac{\chi^{\mu_{1}+\mu_{3}}}{\Gamma (\mu_{1}+\mu_{3}+1)}+$$

$$0.12\frac{\chi^{\mu_{1}+\mu_{3}}}{\Gamma (\mu_{1}+1)\Gamma (\mu_{3}+1)}\frac{\chi^{2\mu_{1}+\mu_{3}}}{\Gamma (2\mu_{1}+\mu_{3}+1)}+864\frac{\chi^{2\mu_{1}}}{\Gamma (2\mu_{1}+1)}+0.384\frac{\chi^{3\mu_{1}}}{\Gamma (3\mu_{1}+1)}+\cdots$$

$$𝔽(\chi )={𝔽}_{0}(\chi )+{𝔽}_{1}(\chi )+{𝔽}_{2}(\chi )+\cdots$$

$$𝔽(\chi )=250+485\frac{\chi^{\mu_{2}}}{\Gamma (\mu_{2}+1)}+0.32\frac{\chi^{\mu_{1}+\mu_{2}}}{\Gamma (\mu_{1}+\mu_{2}+1)}-205\frac{\chi^{\mu_{2}+\mu_{3}}}{\Gamma (\mu_{2}+\mu_{3}+1)}-0.12$$

$$\frac{\chi^{\mu_{1}+\mu_{3}}}{\Gamma (\mu_{1}+1)\Gamma (\mu_{3}+1)}\frac{\chi^{\mu_{1}+\mu_{2}+\mu_{3}}}{\Gamma (\mu_{1}+\mu_{2}+\mu_{3}+1)}-720\frac{\chi^{\mu_{1}+\mu_{2}}}{\Gamma (\mu_{1}+\mu_{2}+1)}-0.32\frac{\chi^{2\mu_{1}+\mu_{2}}}{\Gamma (2\mu_{1}+\mu_{2}+1)}-$$

$$223.1\frac{\chi^{2\mu_{2}}}{\Gamma (2\mu_{2}+1)}+\cdots$$

$$𝔾(\chi )={𝔾}_{0}(\chi )+{𝔾}_{1}(\chi )+{𝔾}_{2}(\chi )+\cdots$$

$$𝔾(\chi )=100-37.5\frac{\chi^{\mu_{3}}}{\Gamma (\mu_{3}+1)}+4.85\frac{\chi^{\mu_{2}+\mu_{3}}}{\Gamma (\mu_{2}+\mu_{3}+1)}+0.0032\frac{\chi^{\mu_{1}+\mu_{2}+\mu_{3}}}{\Gamma (\mu_{1}+\mu_{2}+\mu_{3}+1)}+15$$

$$\frac{\chi^{2\mu_{3}}}{\Gamma (2\mu_{3}+1)}+\cdots$$

$$ℍ(\chi )={ℍ}_{0}(\chi )+{ℍ}_{1}(\chi )+{ℍ}_{2}(\chi )+\cdots$$

$$ℍ(\chi )=50+72.5\frac{\chi^{\mu_{4}}}{\Gamma (\mu_{4}+1)}-7.5\frac{\chi^{\mu_{3}+\mu_{4}}}{\Gamma (\mu_{3}+\mu_{4}+1)}+121.5\frac{\chi^{\mu_{2}+\mu_{4}}}{\Gamma (\mu_{2}+\mu_{4}+1)}+0.08$$

$$\frac{\chi^{\mu_{1}+\mu_{2}+\mu_{4}}}{\Gamma (\mu_{1}+\mu_{2}+\mu_{4}+1)}-14.5\frac{\chi^{2\mu_{4}}}{\Gamma (2\mu_{4}+1)}+\cdots$$

**Application of the YTDM**

By using the YT

$$Y\left(\frac{\partial^{\mu_{1}}𝔼}{\partial \chi^{\mu_{1}}}\right)=Y(ℬ-\beta 𝔼(\chi )𝔾(\chi )-\kappa 𝔼(\chi )).\;Y\left(\frac{\partial^{\mu_{2}}𝔽}{\partial \chi^{\mu_{2}}}\right)=Y(\beta 𝔼(\chi )𝔾(\chi )-(\sigma +\kappa +\alpha )𝔽(\chi )).\;Y\left(\frac{\partial^{\mu_{3}}𝔾}{\partial \chi^{\mu_{3}}}\right)=Y(\alpha 𝔽(\chi )-(\rho +\kappa )𝔾(\chi )).\;Y\left(\frac{\partial^{\mu_{4}}ℍ}{\partial \chi^{\mu_{4}}}\right)=Y(\rho 𝔾(\chi )+\sigma 𝔽(\chi )-\kappa ℍ(\chi )).\;$$ (46)

After we get

$$\frac{1}{\varsigma^{\mu_{1}}}\{ℱ(\varsigma )-\varsigma 𝔼(0)\}=Y(ℬ-\beta 𝔼(\chi )𝔾(\chi )-\kappa 𝔼(\chi )),\;\frac{1}{\varsigma^{\mu_{2}}}\{ℱ(\varsigma )-\varsigma 𝔽(0)\}=Y(\beta 𝔼(\chi )𝔾(\chi )-(\sigma +\kappa +\alpha )𝔽(\chi )),\;\frac{1}{\varsigma^{\mu_{3}}}\{ℱ(\varsigma )-\varsigma 𝔾(0)\}=Y(\alpha 𝔽(\chi )-(\rho +\kappa )𝔾(\chi )),\;\frac{1}{\varsigma^{\mu_{4}}}\{ℱ(\varsigma )-\varsigma ℍ(0)\}=Y(\rho 𝔾(\chi )+\sigma 𝔽(\chi )-\kappa ℍ(\chi )),$$ (47)

$$ℱ(\varsigma )=\varsigma 𝔼(0)+\varsigma^{\mu_{1}}(ℬ-\beta 𝔼(\chi )𝔾(\chi )-\kappa 𝔼(\chi )),\;ℱ(\varsigma )=\varsigma 𝔽(0)+\varsigma^{\mu_{2}}(\beta 𝔼(\chi )𝔾(\chi )-(\sigma +\kappa +\alpha )𝔽(\chi )),\;ℱ(\varsigma )=\varsigma 𝔾(0)+\varsigma^{\mu_{3}}(\alpha 𝔽(\chi )-(\rho +\kappa )𝔾(\chi )),\;ℱ(\varsigma )=\varsigma ℍ(0)+\varsigma^{\mu_{4}}(\rho 𝔾(\chi )+\sigma 𝔽(\chi )-\kappa ℍ(\chi )),$$ (48)

On utilizing the inverse YT

$$𝔼(\chi )=𝔼(0)+Y^{-1}[\varsigma^{\mu_{1}}Y(ℬ)]+Y^{-1}[\varsigma^{q}\left\{Y(-\beta 𝔼(\chi )𝔾(\chi )-\kappa 𝔼(\chi ))\right\}],\;𝔽(\chi )=𝔽(0)+Y^{-1}[\varsigma^{\mu_{2}}\left\{Y(\beta 𝔼(\chi )𝔾(\chi )-(\sigma +\kappa +\alpha )𝔽(\chi ))\right\}],\;𝔾(\chi )=𝔾(0)+Y^{-1}[\varsigma^{\mu_{3}}\left\{Y(\alpha 𝔽(\chi )-(\rho +\kappa )𝔾(\chi ))\right\}],\;ℍ(\chi )=ℍ(0)+Y^{-1}[\varsigma^{\mu_{4}}\left\{Y(\rho 𝔾(\chi )+\sigma 𝔽(\chi )-\kappa ℍ(\chi ))\right\}],$$ (49)

$$𝔼(\chi )=n_{1}+Y^{-1}[\varsigma^{\mu_{1}}Y(ℬ)]+Y^{-1}[\varsigma^{q}\left\{Y(-\beta 𝔼(\chi )𝔾(\chi )-\kappa 𝔼(\chi ))\right\}],\;𝔽(\chi )=n_{2}+Y^{-1}[\varsigma^{\mu_{2}}\left\{Y(\beta 𝔼(\chi )𝔾(\chi )-(\sigma +\kappa +\alpha )𝔽(\chi ))\right\}],\;𝔾(\chi )=n_{3}+Y^{-1}[\varsigma^{\mu_{3}}\left\{Y(\alpha 𝔽(\chi )-(\rho +\kappa )𝔾(\chi ))\right\}],\;ℍ(\chi )=n_{4}+Y^{-1}[\varsigma^{\mu_{4}}\left\{Y(\rho 𝔾(\chi )+\sigma 𝔽(\chi )-\kappa ℍ(\chi ))\right\}].\;$$ (50)

We may get the series form solution as

$$𝔼(\chi )=\sum_{m=0}^{\infty }{𝔼}_{m}(\chi ),𝔽(\chi )=\sum_{m=0}^{\infty }{𝔽}_{m}(\chi ),𝔾(\chi )=\sum_{m=0}^{\infty }{𝔾}_{m}(\chi ),ℍ(\chi )=\sum_{m=0}^{\infty }{ℍ}_{m}(\chi ).$$ (51)

The nonlinear term is broken down as $𝔼(\chi )𝔾(\chi )=\sum_{m=0}^{\infty }{𝒜}_{m}.$

$$\sum_{m=0}^{\infty }{𝔼}_{m}(\chi )=n_{1}+ℬ\frac{\chi^{\mu_{1}}}{\Gamma (\mu_{1}+1)}+Y^{-1}[\varsigma^{q}Y[-\beta (\sum_{m=0}^{\infty }{𝒜}_{m})-\kappa 𝔼(\chi )]],\;\sum_{m=0}^{\infty }{𝔽}_{m}(\chi )=𝔽(0)+Y^{-1}[\varsigma^{q}Y[\beta (\sum_{m=0}^{\infty }{𝒜}_{m})-(\sigma +\kappa +\alpha )𝔽(\chi )]],\;\sum_{m=0}^{\infty }{𝔾}_{m}(\chi )=𝔾(0)+Y^{-1}[\varsigma^{q}Y[\alpha 𝔽(\chi )-(\rho +\kappa )𝔾(\chi )]],\;\sum_{m=0}^{\infty }{ℍ}_{m}(\chi )=ℍ(0)+Y^{-1}[\varsigma^{q}Y[\rho 𝔾(\chi )+\sigma 𝔽(\chi )-\kappa ℍ(\chi )]],\;\sum_{m=0}^{\infty }{𝔼}_{m}(\chi )=n_{1}+ℬ\frac{\chi^{\mu_{1}}}{\Gamma (\mu_{1}+1)}+Y^{-1}[\varsigma^{q}Y[-\beta (\sum_{m=0}^{\infty }{𝒜}_{m})-\kappa 𝔼(\chi )]],\;\sum_{m=0}^{\infty }{𝔽}_{m}(\chi )=n_{2}+Y^{-1}[\varsigma^{q}Y[\beta (\sum_{m=0}^{\infty }{𝒜}_{m})-(\sigma +\kappa +\alpha )𝔽(\chi )]],\;\sum_{m=0}^{\infty }{𝔾}_{m}(\chi )=n_{3}+Y^{-1}[\varsigma^{q}Y[\alpha 𝔽(\chi )-(\rho +\kappa )𝔾(\chi )]],\;\sum_{m=0}^{\infty }{ℍ}_{m}(\chi )=n_{4}+Y^{-1}[\varsigma^{q}Y[\rho 𝔾(\chi )+\sigma 𝔽(\chi )-\kappa ℍ(\chi )]].$$ (52)

For numerical results, we used the following Table (1) values of parameters are considered from [10]. By comparing both sides, we may get


$${𝔼}_{0}(\chi )=600+0.32\frac{\chi^{\mu_{1}}}{\Gamma (\mu_{1}+1)},$$

$${𝔼}_{0}(\chi )=250,$$

$${𝔼}_{0}(\chi )=100,$$

$${𝔼}_{0}(\chi )=50.$$

On *m* = 0


$${𝔼}_{1}(\chi )=-720\frac{\chi^{\mu_{1}}}{\Gamma (\mu_{1}+1)}-0.32\frac{\chi^{2\mu_{1}}}{\Gamma (2\mu_{1}+1)},$$

$${𝔽}_{1}(\chi )=485\frac{\chi^{\mu_{2}}}{\Gamma (\mu_{2}+1)}+0.32\frac{\chi^{\mu_{1}+\mu_{2}}}{\Gamma (\mu_{1}+\mu_{2}+1)},$$

$${𝔾}_{1}(\chi )=-37.5\frac{\chi^{\mu_{3}}}{\Gamma (\mu_{3}+1)},$$

$${ℍ}_{1}(\chi )=72.5\frac{\chi^{\mu_{4}}}{\Gamma (\mu_{4}+1)}.$$

On *m* = 1


$${𝔼}_{2}(\chi )=205\frac{\chi^{\mu_{1}+\mu_{3}}}{\Gamma (\mu_{1}+\mu_{3}+1)}+0.12\frac{\chi^{\mu_{1}+\mu_{3}}}{\Gamma (\mu_{1}+1)\Gamma (\mu_{3}+1)}\frac{\chi^{2\mu_{1}+\mu_{3}}}{\Gamma (2\mu_{1}+\mu_{3}+1)}+864\frac{\chi^{2\mu_{1}}}{\Gamma (2\mu_{1}+1)}$$

$$+0.384\frac{\chi^{3\mu_{1}}}{\Gamma (3\mu_{1}+1)},$$

$${𝔽}_{2}(\chi )=-205\frac{\chi^{\mu_{2}+\mu_{3}}}{\Gamma (\mu_{2}+\mu_{3}+1)}-0.12\frac{\chi^{\mu_{1}+\mu_{3}}}{\Gamma (\mu_{1}+1)\Gamma (\mu_{3}+1)}\frac{\chi^{\mu_{1}+\mu_{2}+\mu_{3}}}{\Gamma (\mu_{1}+\mu_{2}+\mu_{3}+1)}-720$$

$$\frac{\chi^{\mu_{1}+\mu_{2}}}{\Gamma (\mu_{1}+\mu_{2}+1)}-0.32\frac{\chi^{2\mu_{1}+\mu_{2}}}{\Gamma (2\mu_{1}+\mu_{2}+1)}-223.1\frac{\chi^{2\mu_{2}}}{\Gamma (2\mu_{2}+1)},$$

$${𝔾}_{2}(\chi )=4.85\frac{\chi^{\mu_{2}+\mu_{3}}}{\Gamma (\mu_{2}+\mu_{3}+1)}+0.0032\frac{\chi^{\mu_{1}+\mu_{2}+\mu_{3}}}{\Gamma (\mu_{1}+\mu_{2}+\mu_{3}+1)}+15\frac{\chi^{2\mu_{3}}}{\Gamma (2\mu_{3}+1)},$$

$${ℍ}_{2}(\chi )=-7.5\frac{\chi^{\mu_{3}+\mu_{4}}}{\Gamma (\mu_{3}+\mu_{4}+1)}+121.5\frac{\chi^{\mu_{2}+\mu_{4}}}{\Gamma (\mu_{2}+\mu_{4}+1)}+0.08\frac{\chi^{\mu_{1}+\mu_{2}+\mu_{4}}}{\Gamma (\mu_{1}+\mu_{2}+\mu_{4}+1)}-14.5$$

$$\frac{\chi^{2\mu_{4}}}{\Gamma (2\mu_{4}+1)},$$

Finally, the approximate solution is derived as


$$𝔼(\chi )={𝔼}_{0}(\chi )+{𝔼}_{1}(\chi )+{𝔼}_{2}(\chi )+\cdots$$

$$𝔼(\chi )=600+0.32\frac{\chi^{\mu_{1}}}{\Gamma (\mu_{1}+1)}-720\frac{\chi^{\mu_{1}}}{\Gamma (\mu_{1}+1)}-0.32\frac{\chi^{2\mu_{1}}}{\Gamma (2\mu_{1}+1)}+205\frac{\chi^{\mu_{1}+\mu_{3}}}{\Gamma (\mu_{1}+\mu_{3}+1)}+$$

$$0.12\frac{\chi^{\mu_{1}+\mu_{3}}}{\Gamma (\mu_{1}+1)\Gamma (\mu_{3}+1)}\frac{\chi^{2\mu_{1}+\mu_{3}}}{\Gamma (2\mu_{1}+\mu_{3}+1)}+864\frac{\chi^{2\mu_{1}}}{\Gamma (2\mu_{1}+1)}+0.384\frac{\chi^{3\mu_{1}}}{\Gamma (3\mu_{1}+1)}+\cdots$$

$$𝔽(\chi )={𝔽}_{0}(\chi )+{𝔽}_{1}(\chi )+{𝔽}_{2}(\chi )+\cdots$$

$$𝔽(\chi )=250+485\frac{\chi^{\mu_{2}}}{\Gamma (\mu_{2}+1)}+0.32\frac{\chi^{\mu_{1}+\mu_{2}}}{\Gamma (\mu_{1}+\mu_{2}+1)}-205\frac{\chi^{\mu_{2}+\mu_{3}}}{\Gamma (\mu_{2}+\mu_{3}+1)}-0.12$$

$$\frac{\chi^{\mu_{1}+\mu_{3}}}{\Gamma (\mu_{1}+1)\Gamma (\mu_{3}+1)}\frac{\chi^{\mu_{1}+\mu_{2}+\mu_{3}}}{\Gamma (\mu_{1}+\mu_{2}+\mu_{3}+1)}-720\frac{\chi^{\mu_{1}+\mu_{2}}}{\Gamma (\mu_{1}+\mu_{2}+1)}-0.32\frac{\chi^{2\mu_{1}+\mu_{2}}}{\Gamma (2\mu_{1}+\mu_{2}+1)}-$$

$$223.1\frac{\chi^{2\mu_{2}}}{\Gamma (2\mu_{2}+1)}+\cdots$$

$$𝔾(\chi )={𝔾}_{0}(\chi )+{𝔾}_{1}(\chi )+{𝔾}_{2}(\chi )+\cdots$$

$$𝔾(\chi )=100-37.5\frac{\chi^{\mu_{3}}}{\Gamma (\mu_{3}+1)}+4.85\frac{\chi^{\mu_{2}+\mu_{3}}}{\Gamma (\mu_{2}+\mu_{3}+1)}+0.0032\frac{\chi^{\mu_{1}+\mu_{2}+\mu_{3}}}{\Gamma (\mu_{1}+\mu_{2}+\mu_{3}+1)}+15$$

$$\frac{\chi^{2\mu_{3}}}{\Gamma (2\mu_{3}+1)}+\cdots$$

$$ℍ(\chi )={ℍ}_{0}(\chi )+{ℍ}_{1}(\chi )+{ℍ}_{2}(\chi )+\cdots$$

$$ℍ(\chi )=50+72.5\frac{\chi^{\mu_{4}}}{\Gamma (\mu_{4}+1)}-7.5\frac{\chi^{\mu_{3}+\mu_{4}}}{\Gamma (\mu_{3}+\mu_{4}+1)}+121.5\frac{\chi^{\mu_{2}+\mu_{4}}}{\Gamma (\mu_{2}+\mu_{4}+1)}+0.08$$

$$\frac{\chi^{\mu_{1}+\mu_{2}+\mu_{4}}}{\Gamma (\mu_{1}+\mu_{2}+\mu_{4}+1)}-14.5\frac{\chi^{2\mu_{4}}}{\Gamma (2\mu_{4}+1)}+\cdots$$

## 8 Results and discussion

**Fig 2:**
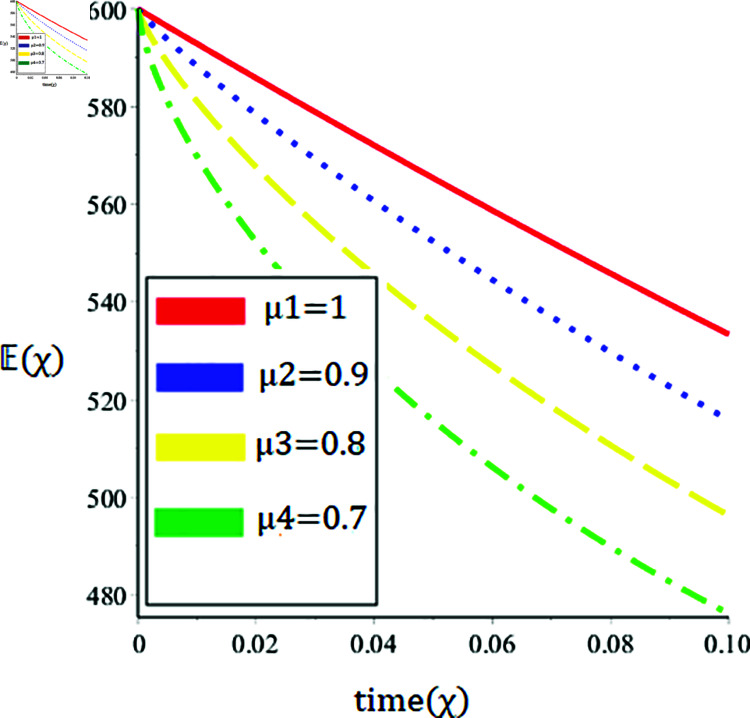
Nature of the susceptible population 𝔼(χ) in terms of HPTM for a time χ (year) at various values of μ.

**Fig 3:**
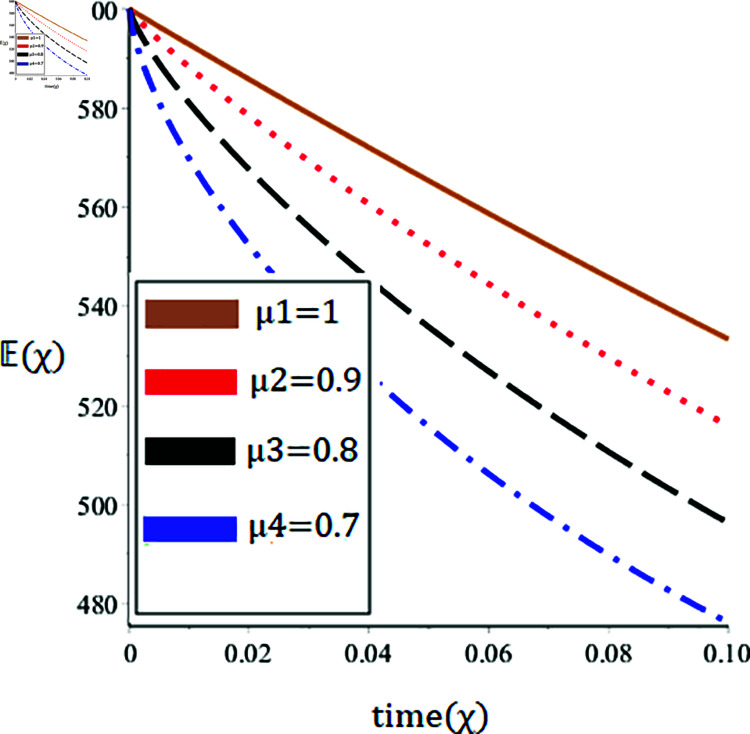
Nature of the susceptible population 𝔼(χ) in terms of YTDM for a time χ (year) at various values of μ.

**Fig 4:**
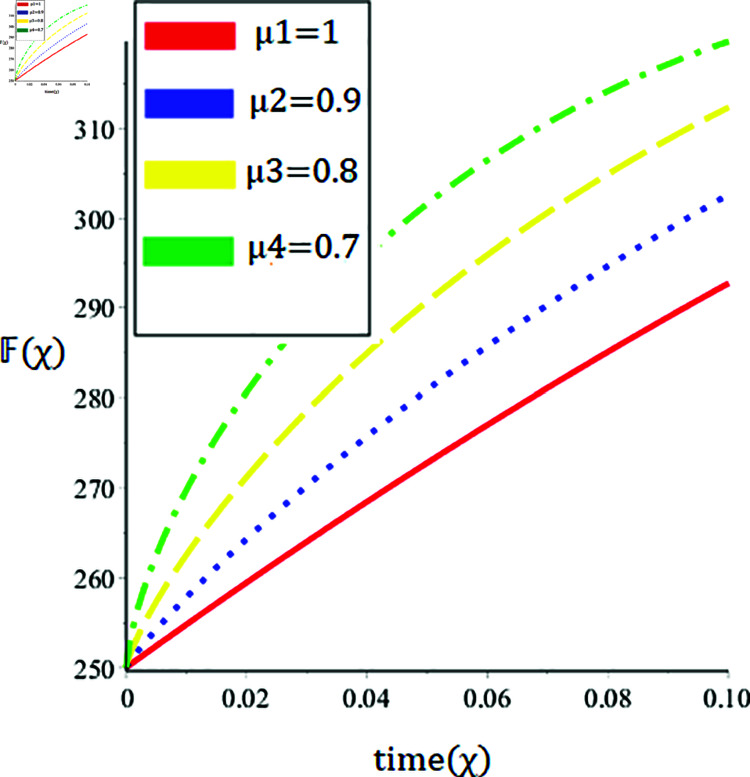
Nature of the exposed population 𝔽(χ) in terms of HPTM for a time χ (year) at various values of μ.

**Fig 5:**
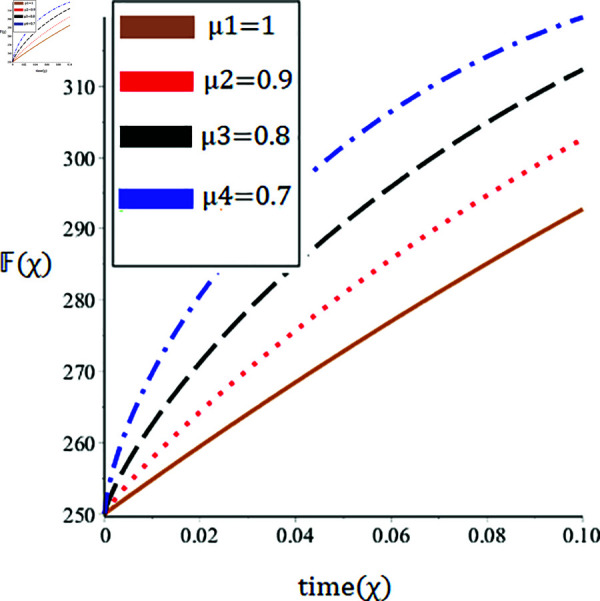
Nature of the exposed population 𝔽(χ) in terms of YTDM for a time χ (year) at various values of μ.

**Fig 6:**
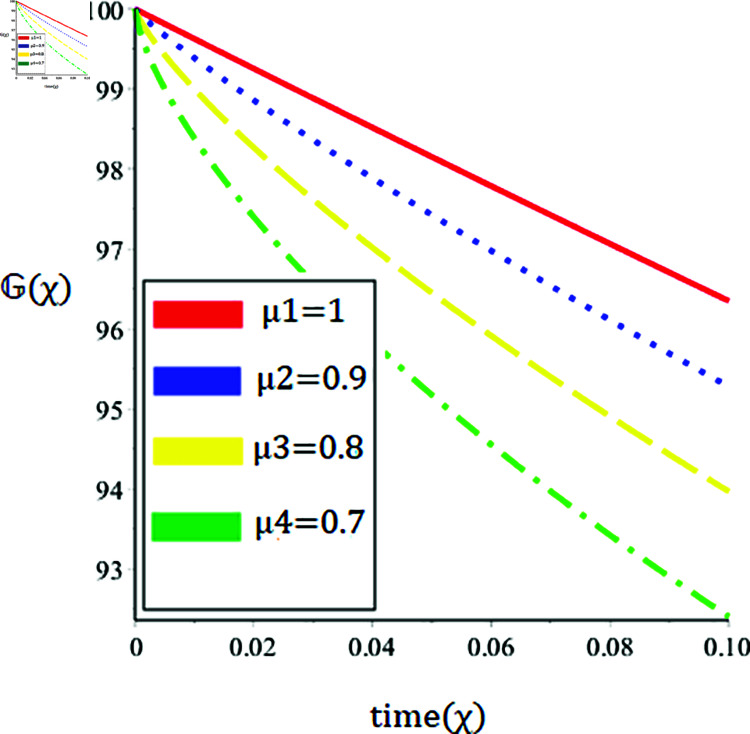
Nature of the infectious population 𝔾(χ) in terms of HPTM for a time χ (year) at various values of μ.

**Fig 7:**
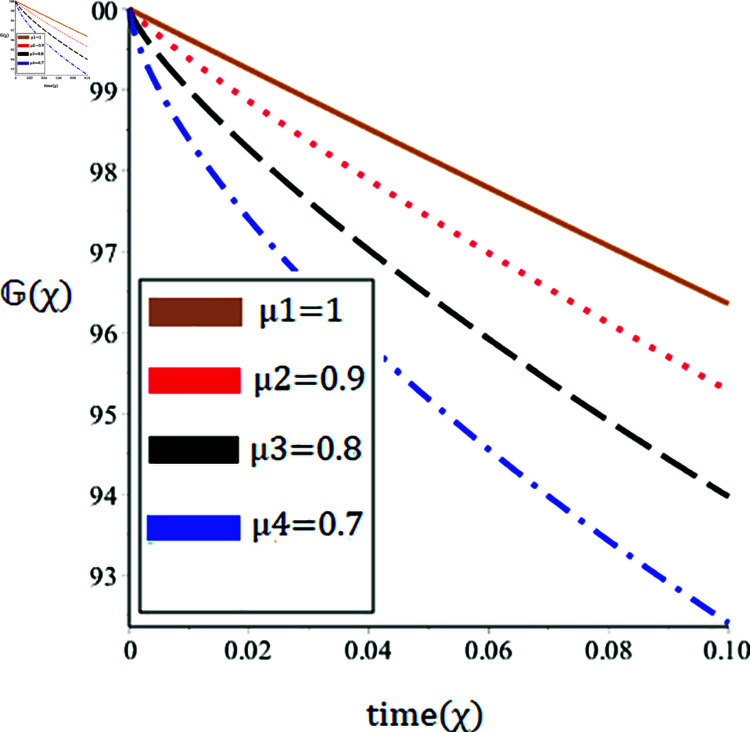
Nature of the infectious population 𝔾(χ) in terms of YTDM for a time χ (year) at various values of μ.

**Fig 8:**
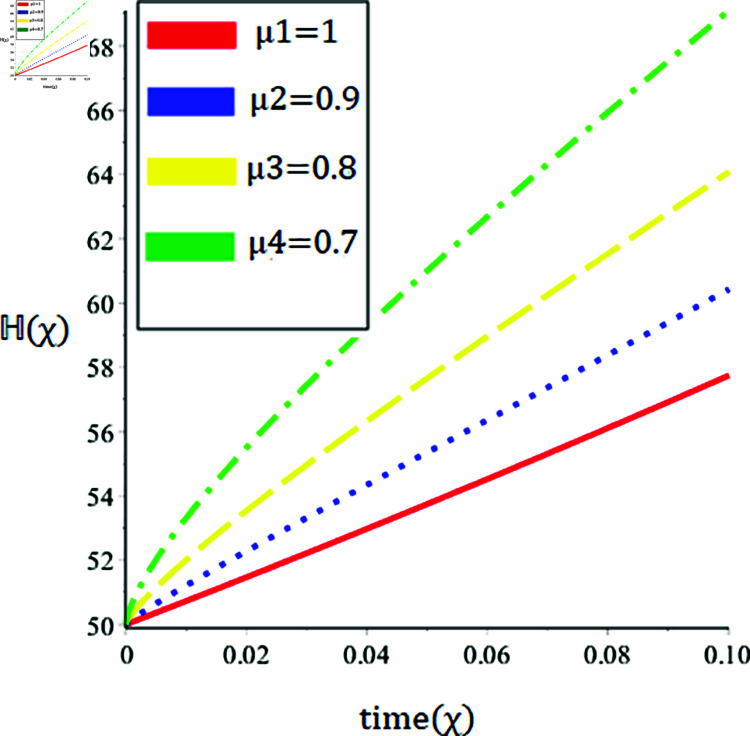
Nature of the recovered population ℍ(χ) in terms of HPTM for a time χ (year) at various values of μ.

**Fig 9:**
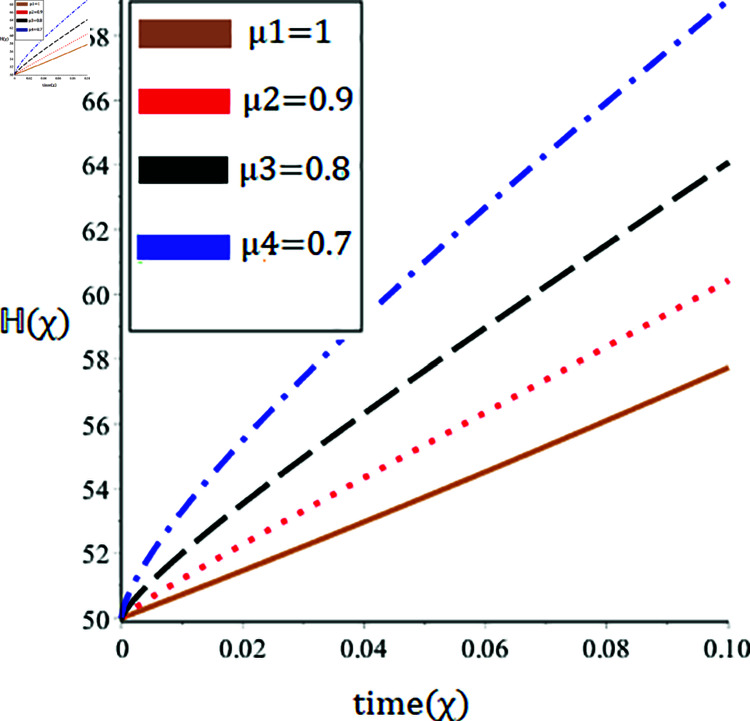
Nature of the recovered population ℍ(χ) in terms of YTDM for a time χ (year) at various values of μ.

The current paper presents the numerical solution of the measles epidemic model using a nonlinear differential equation system. The behaviour of the model is represented by looking at solutions up to a third-order series. From the plot, we see that when the order is smaller faster the decay of susceptible population 𝔼(χ), this behavior can be observed from the Fig 2 and Fig 3. From Fig 4 and Fig 5, we see that the exposed class significantly grows at smaller order with the passage of time. Similarly from Fig 6 and Fig 7, one can observes that smaller the fractional order fastest the decaying process of the infected class with the passage of time. In the Fig 8 and Fig 9, we may observe that the recovered class grows more rapidly on the smaller order of the differentiation over time. As demonstrated in all figures, evaluation is conducted for a range of μ values in order to conduct a reliable research. In comparison to ordinary derivatives, we find that the fractional order SEIR epidemic model has a greater degree of freedom. HPTM and YTDM are used to establish the numerical results of SEIR population for various values of μ in Tables 2–9. Outstanding responses from the compartments in the suggested model are obtained by using non-integer values for the fractional parameter. Another noteworthy thing to keep in mind is that we choose a short time interval because we assumed relatively small initial values. The initial values of the data are taken large for long intervals of time to prevent negative patterns in the population. The solution converges to a steady state for a range of μ values. It provides faster convergence by decreasing the fractional values of μ. The figures provided demonstrate that the anticipated model has a higher degree of flexibility and is significantly dependent on the order. The behaviour that has been described serves as a case study of the capability and efficacy of the suggested solution strategies. Moreover, the fractional operator under investigation provides more interesting outcomes for examining and projecting the future of the model under consideration. The current study may contribute to our understanding of the deadly virus because epidemic models depend heavily on genetic characteristics.

**Table 2:** Table demonstrating the behavior of susceptible population 𝔼(χ) at various values of μ in terms of HPTM.

χ	$\mu_{1}=1$	$\mu_{1}=0.99$	$\mu_{1}=0.98$	$\mu_{1}=0.97$
	$\mu_{2}=1$	$\mu_{2}=0.99$	$\mu_{2}=0.98$	$\mu_{2}=0.97$
	$\mu_{3}=1$	$\mu_{3}=0.99$	$\mu_{3}=0.98$	$\mu_{3}=0.97$
	$\mu_{4}=1$	$\mu_{4}=0.99$	$\mu_{4}=0.98$	$\mu_{4}=0.97$
0.0	600	600	600	600
0.1	533.375504	531.745137	530.087596	528.403183
0.3	432.189408	430.741439	429.332238	427.964436
0.5	373.758000	374.109309	374.560208	375.113438
0.7	358.086272	360.927343	363.902026	367.011849
0.9	385.179216	390.730285	396.419559	402.246714

**Table 3:** Table demonstrating the behavior of susceptible population 𝔼(χ) at various values of μ in terms of YTDM.

χ	$\mu_{1}=1$	$\mu_{1}=0.99$	$\mu_{1}=0.98$	$\mu_{1}=0.97$
	$\mu_{2}=1$	$\mu_{2}=0.99$	$\mu_{2}=0.98$	$\mu_{2}=0.97$
	$\mu_{3}=1$	$\mu_{3}=0.99$	$\mu_{3}=0.98$	$\mu_{3}=0.97$
	$\mu_{4}=1$	$\mu_{4}=0.99$	$\mu_{4}=0.98$	$\mu_{4}=0.97$
0.0	600	600	600	600
0.1	533.375504	531.745137	530.087596	528.403183
0.3	432.189408	430.741439	429.332238	427.964436
0.5	373.758000	374.109309	374.560208	375.113438
0.7	358.086272	360.927343	363.902026	367.011849
0.9	385.179216	390.730285	396.419559	402.246714

**Table 4:** Table demonstrating the behavior of exposed population 𝔽(χ) at various values of μ in terms of HPTM.

χ	$\mu_{1}=1$	$\mu_{1}=0.99$	$\mu_{1}=0.98$	$\mu_{1}=0.97$
	$\mu_{2}=1$	$\mu_{2}=0.99$	$\mu_{2}=0.98$	$\mu_{2}=0.97$
	$\mu_{3}=1$	$\mu_{3}=0.99$	$\mu_{3}=0.98$	$\mu_{3}=0.97$
	$\mu_{4}=1$	$\mu_{4}=0.99$	$\mu_{4}=0.98$	$\mu_{4}=0.97$
0.0	250	250	250	250
0.1	292.761006	293.717449	294.683122	295.657274
0.3	343.847380	343.988639	344.070910	344.091313
0.5	349.015833	347.027708	344.924160	342.702421
0.7	308.261886	303.640147	298.875966	293.968000
0.9	221.581060	214.255746	206.791833	199.190007

**Table 5:** Table demonstrating the behavior of exposed population 𝔽(χ) at various values of μ in terms of YTDM.

χ	$\mu_{1}=1$	$\mu_{1}=0.99$	$\mu_{1}=0.98$	$\mu_{1}=0.97$
	$\mu_{2}=1$	$\mu_{2}=0.99$	$\mu_{2}=0.98$	$\mu_{2}=0.97$
	$\mu_{3}=1$	$\mu_{3}=0.99$	$\mu_{3}=0.98$	$\mu_{3}=0.97$
	$\mu_{4}=1$	$\mu_{4}=0.99$	$\mu_{4}=0.98$	$\mu_{4}=0.97$
0.0	250	250	250	250
0.1	292.761006	293.717449	294.683122	295.657274
0.3	343.847380	343.988639	344.070910	344.091313
0.5	349.015833	347.027708	344.924160	342.702421
0.7	308.261886	303.640147	298.875966	293.968000
0.9	221.581060	214.255746	206.791833	199.190007

**Table 6:** Table demonstrating the behavior of infectious population 𝔾(χ) at various values of μ in terms of HPTM.

χ	$\mu_{1}=1$	$\mu_{1}=0.99$	$\mu_{1}=0.98$	$\mu_{1}=0.97$
	$\mu_{2}=1$	$\mu_{2}=0.99$	$\mu_{2}=0.98$	$\mu_{2}=0.97$
	$\mu_{3}=1$	$\mu_{3}=0.99$	$\mu_{3}=0.98$	$\mu_{3}=0.97$
	$\mu_{4}=1$	$\mu_{4}=0.99$	$\mu_{4}=0.98$	$\mu_{4}=0.97$
0.0	100	100	100	100
0.1	96.349250	96.252373	96.153319	96.052057
0.3	89.643264	89.497868	89.351738	89.204917
0.5	83.731316	83.602821	83.475466	83.349316
0.7	78.613432	78.534546	78.458229	78.384533
0.9	74.289638	74.278402	74.270555	74.266118

**Table 7:** Table demonstrating the behavior of infectious population 𝔾(χ) at various values of μ in terms of YTDM.

χ	$\mu_{1}=1$	$\mu_{1}=0.99$	$\mu_{1}=0.98$	$\mu_{1}=0.97$
	$\mu_{2}=1$	$\mu_{2}=0.99$	$\mu_{2}=0.98$	$\mu_{2}=0.97$
	$\mu_{3}=1$	$\mu_{3}=0.99$	$\mu_{3}=0.98$	$\mu_{3}=0.97$
	$\mu_{4}=1$	$\mu_{4}=0.99$	$\mu_{4}=0.98$	$\mu_{4}=0.97$
0.0	100	100	100	100
0.1	96.349250	96.252373	96.153319	96.052057
0.3	89.643264	89.497868	89.351738	89.204917
0.5	83.731316	83.602821	83.475466	83.349316
0.7	78.613432	78.534546	78.458229	78.384533
0.9	74.289638	74.278402	74.270555	74.266118

**Table 8:** Table demonstrating the behavior of recovered population ℍ(χ) at various values of μ in terms of HPTM.

χ	$\mu_{1}=1$	$\mu_{1}=0.99$	$\mu_{1}=0.98$	$\mu_{1}=0.97$
	$\mu_{2}=1$	$\mu_{2}=0.99$	$\mu_{2}=0.98$	$\mu_{2}=0.97$
	$\mu_{3}=1$	$\mu_{3}=0.99$	$\mu_{3}=0.98$	$\mu_{3}=0.97$
	$\mu_{4}=1$	$\mu_{4}=0.99$	$\mu_{4}=0.98$	$\mu_{4}=0.97$
0.0	50	50	50	50
0.1	57.746263	57.979355	58.219579	58.467171
0.3	76.216610	76.766338	77.328063	77.902059
0.5	98.657916	99.470110	100.295655	101.134718
0.7	125.070823	126.094805	127.130693	128.178485
0.9	155.455970	156.631639	157.815398	159.007056

**Table 9:** Table demonstrating the behavior of recovered population ℍ(χ) at various values of μ in terms of YTDM.

χ	$\mu_{1}=1$	$\mu_{1}=0.99$	$\mu_{1}=0.98$	$\mu_{1}=0.97$
	$\mu_{2}=1$	$\mu_{2}=0.99$	$\mu_{2}=0.98$	$\mu_{2}=0.97$
	$\mu_{3}=1$	$\mu_{3}=0.99$	$\mu_{3}=0.98$	$\mu_{3}=0.97$
	$\mu_{4}=1$	$\mu_{4}=0.99$	$\mu_{4}=0.98$	$\mu_{4}=0.97$
0.0	50	50	50	50
0.1	57.746263	57.979355	58.219579	58.467171
0.3	76.216610	76.766338	77.328063	77.902059
0.5	98.657916	99.470110	100.295655	101.134718
0.7	125.070823	126.094805	127.130693	128.178485
0.9	155.455970	156.631639	157.815398	159.007056

## 9 Conclusion

Finding the numerical solutions for the fractional SIER model of disease is necessary due to the increasing number of disease models. A system of coupled, non-linear ordinary differential equations describes the population dynamics during the illness in the model. To the best of the author’s knowledge, no precise solution for this model can be found in the literature. This work suggests comparing two unique approaches for the fractional SEIR epidemic model namely the YTDM and HPTM. The results are found as a quickly convergent series of solutions. Additionally, numerical simulations are displayed along with the compression for a variety of values of a. Tables and graphs show how the fractional parameter affected our found solutions. As compared to regular derivatives, it is important to note that fractional derivatives exhibit notable modifications and memory effects. By incorporating fractional calculus into mathematical models, researchers can gain deeper insights into the complex behaviors of biological systems and develop innovative approaches to address key challenges in biomedical research and health care. According to the authors, biologists will find this study to be more beneficial and efficient. Moreover, nonlinear fractional-order mathematical models of infectious diseases such as hepatitis, TB, and Ebola can be studied using the same techniques. Readers can employ hybrid transforms merging with our proposed schemes as a future study direction to attain better outcomes. The addition of more operators will therefore be highly desired in the future, especially in light of the benefits of the current operator.

## References

[1] Allen LJ, Brauer F, Van den Driessche P, Wu J. Mathematical epidemiology, vol. 1945. Berlin: Springer; 2008.

[2] Ma Z, Li J. Dynamical modeling and analysis of epidemics. 2009.

[3] Murray JD. Mathematical biology: I. An introduction, vol. 17. Springer; 2007.

[4] Sontag ED. Lecture notes on mathematical systems biology. New Brunswick, New Jersey: Rutgers University; 2011.

[5] Kermack WO, McKendrick AG. A contribution to the mathematical theory of epidemics. Proc Roy Soc Lond Ser A Contain Papers Math Phys Charact. 1927;115(772):700–21.

[6] AL-Smadi M, Gumah G. On the homotopy analysis method for fractional SEIR epidemic model. RJASET. 2014;7(18):3809–20. doi: 10.19026/rjaset.7.738

[7] El-Sheikh MMA, El-Marouf SAA. On stability and bifurcation of solutions of an SEIR epidemic model with vertical transmission. Int J Math Math Sci. 2004;2004(56):2971–87. doi: 10.1155/s0161171204310380

[8] Ali Z, Rabiei F, Shah K, Khodadadi T. Modeling and analysis of novel covid-19 under fractal-fractional derivative with case study of Malaysia. Fractals. 2021;29(01):2150020. doi: 10.1142/s0218348x21500201

[9] Wang Y, Jurrat B, Ejaz M, Azeem M, Elashiry MI. Existence and uniqueness of well-posed fractional boundary value problem. PLoS One. 2024;19(5):e0303848. doi: 10.1371/journal.pone.0303848 38805425 PMC11132467

[10] Momoh AA, Ibrahim MO, Uwanta IJ, Manga SB. Mathematical model for control of measles epidemiology. Int J of Pure and Appl Math. 2013;87(5). doi: 10.12732/ijpam.v87i5.4

[11] Zhang N, Addai E, Zhang L, Ngungu M, Marinda E, Asamoah JKK. Fractional modeling and numerical simulation for unfolding Marburg–Monkeypox virus co-infection transmission. Fractals. 2023;31(07). doi: 10.1142/s0218348x2350086x

[12] Naik PA, Yeolekar BM, Qureshi S, Manhas N, Ghoreishi M, Yeolekar M, et al. Global analysis of a fractional-order hepatitis B virus model under immune response in the presence of cytokines. Adv Theory Sims. 2024;7(12). doi: 10.1002/adts.202400726

[13] Ganie AH, Khan A, Alhamzi G, Saeed AM, Jeelani M begum. A new solution of the nonlinear fractional logistic differential equations utilizing efficient techniques. AIP Advances. 2024;14(3). doi: 10.1063/5.0197704

[14] Naik PA, Zu J, Naik M. Stability analysis of a fractional-order cancer model with chaotic dynamics. Int J Biomath. 2021;14(06):2150046. doi: 10.1142/s1793524521500467

[15] Higazy M, Hijaz H, Ganie AH, Botmart T, El-Mesady A. Theoretical analysis and computational modeling of nonlinear fractional-order two predators model. Results Phys. 2021;2021:105139.

[16] Moaddy K, Freihat A, Al-Smadi M, Abuteen E, Hashim I. Numerical investigation for handling fractional-order Rabinovich–Fabrikant model using the multistep approach. Soft Comput. 2016;22(3):773–82. doi: 10.1007/s00500-016-2378-5

[17] Ahmed N, Shaikh TS, Rafiq M, Eldin SM, Ganie AH, Ali M, et al. Structure preserving splitting techniques for Ebola reaction–diffusion epidemic system. Fractals. 2023;31(02). doi: 10.1142/s0218348x23400418

[18] Podlubny I. Fractional differential equations: an introduction to fractional derivatives, fractional differential equations, to methods of their solution and some of their applications. Elsevier; 1998.

[19] Baleanu D, Diethelm K, Scalas E, Trujillo JJ. Fractional calculus: models and numerical methods, vol. 3. World Scientific; 2012.

[20] Liu F, Zhuang P, Liu Q. Numerical methods of fractional partial differential equations and applications. 2015.

[21] Ross B. The development of fractional calculus 1695–1900. Historia Mathematica. 1977;4(1):75–89. doi: 10.1016/0315-0860(77)90039-8

[22] Oldham KB, Spanier J. The fractional calculus. New York: Academic Press; 1974.

[23] Abdelrazec A, Pelinovsky D. Convergence of the Adomian decomposition method for initial-value problems. Numer Methods Partial Diff Eq. 2011;27(4):749–66. doi: 10.1002/num.20549

[24] Odibat Z. Approximations of fractional integrals and Caputo fractional derivatives. Appl Math Comput. 2006;178(2):527–33. doi: 10.1016/j.amc.2005.11.072

[25] Luchko YURII, Gorenflo R. An operational method for solving fractional differential equations with the Caputo derivatives. Acta Math Vietnam. 1999;24(2):207–33.

[26] Luchko YuF, Srivastava HM. The exact solution of certain differential equations of fractional order by using operational calculus. Comput Math Appl. 1995;29(8):73–85. doi: 10.1016/0898-1221(95)00031-s

[27] Momani S, Freihat A, Al-Smadi M. Analytical study of fractional-order multiple chaotic FitzHugh-Nagumo neurons model using multistep generalized differential transform method. Abstr Appl Anal. 2014;2014(1):276279.

[28] Das S. Functional fractional calculus, vol. 1. Berlin: Springer; 2011.

[29] Ganie AH, Alharthi NS, Khan A, Saeed AM, Shah MA, Mallik S. The series solutions of fractional foam drainage and fractional modified regularized long wave problems. J Inequal Appl. 2024;2024(1). doi: 10.1186/s13660-024-03227-w

[30] AlBaidani MM, Aljuaydi F, Alsubaie SAF, Ganie AH, Khan A. Computational and numerical analysis of the Caputo-type fractional nonlinear dynamical systems via novel transform. Fract Fraction. 2024;8(12):708.

[31] Fadhal E, Ganie AH, Alharthi NS, Khan A, Fathima D, Elamin AEAMA. On the analysis and deeper properties of the fractional complex physical models pertaining to nonsingular kernels. Sci Rep. 2024;14(1):22182. doi: 10.1038/s41598-024-69500-6 39333163 PMC11436656

[32] Alharbi R, Jan R, Alyobi S, Altayeb Y, Khan Z. Mathematical modeling and stability analysis of the dynamics of monkeypox via fractional-calculus. Fractals. 2022;30(10). doi: 10.1142/s0218348x22402666

[33] Addai E, Zhang L, Asamoah JKK, Essel JF. A fractional order age-specific smoke epidemic model. Appl Math Model. 2023;119:99–118. doi: 10.1016/j.apm.2023.02.019

[34] Mohammad M, Trounev A, Cattani C. The dynamics of COVID-19 in the UAE based on fractional derivative modeling using Riesz wavelets simulation. Adv Differ Equ. 2021;2021(1):115. doi: 10.1186/s13662-021-03262-7 33623526 PMC7893134

[35] Zhang L, Addai E, Ackora-Prah J, Arthur YD, Asamoah JKK. Fractional-order Ebola-Malaria coinfection model with a focus on detection and treatment rate. Comput Math Methods Med. 2022;2022:6502598. doi: 10.1155/2022/6502598 36158132 PMC9507665

[36] Haq F, Shah K, ur Rahman G, Shahzad M. Numerical solution of fractional order smoking model via laplace Adomian decomposition method. Alexandria Eng J. 2018;57(2):1061–9. doi: 10.1016/j.aej.2017.02.015

[37] Yavuz M, Sulaiman TA, Usta F, Bulut H. Analysis and numerical computations of the fractional regularized long-wave equation with damping term. Math Methods in App Sciences. 2020;44(9):7538–55. doi: 10.1002/mma.6343

[38] Kumar S, Ghosh S, Samet B, Goufo EFD. An analysis for heat equations arises in diffusion process using new Yang-Abdel-Aty-Cattani fractional operator. Math Methods in App Sciences. 2020;43(9):6062–80. doi: 10.1002/mma.6347

[39] Doungmo Goufo EF, Kumar S, Mugisha SB. Similarities in a fifth-order evolution equation with and with no singular kernel. Chaos, Solitons Fractals. 2020;130:109467. doi: 10.1016/j.chaos.2019.109467

[40] AlBaidani MM, Ganie AH, Aljuaydi F, Khan A. Application of analytical techniques for solving fractional physical models arising in applied sciences. Fractal Fract. 2023;7(8):584. doi: 10.3390/fractalfract7080584

[41] De la Sen M. About robust stability of Caputo linear fractional dynamic systems with time delays through fixed point theory. Fixed Point Theory Appl. 2011;2011(1). doi: 10.1155/2011/867932

[42] De la Sen M. Positivity and stability of the solutions of Caputo fractional linear time-invariant systems of any order with internal point delays. Abstract Appl Anal. 2011;2011(1):161246.

[43] Arqub OA, El-Ajou A. Solution of the fractional epidemic model by homotopy analysis method. J King Saud Univ - Sci. 2013;25(1):73–81. doi: 10.1016/j.jksus.2012.01.003

[44] Podlubny I, Kacenak M. Isoclinal matrices and numerical solution of fractional differential equations. In: 2001 European Control Conference (ECC). 2001. p. 1467–70. doi: 10.23919/ecc.2001.7076125

[45] Yang XJ, Baleanu D, Srivastava HM. Local fractional integral transforms and their applications. Academic Press; 2015.

